# 1,3-Dimethyl-5,6,7,8-tetra­hydro-4*H*-cyclo­hepta­[*c*]thio­phene-4,8-dione

**DOI:** 10.1107/S1600536810047331

**Published:** 2010-11-20

**Authors:** Lijuan Yu, Yinghui Yin, Xiaole Zhou, Renjie Li, Tianyou Peng

**Affiliations:** a College of Chemistry and Molecular Science, Wuhan University, Wuhan 430072, People’s Republic of China

## Abstract

In the title compound, C_11_H_12_O_2_S, the C and S atoms of the central thio­phene and the methyl groups, and the two carbonyl groups of the cyclo­hepta­nedione are almost coplanar [maximum deviation from the mean plane = 0.221 (2) Å]. The packing is stabilized by π–π inter­actions between the conjugated thio­phenes, the shortest centroid–centroid distance between thio­phene rings being 3.9759 (10) Å.

## Related literature

The title compound was obtained as the product of our ongoing research of conjugated thio­phenes for electronic devices and dye-sensitized solar cells (DSSCs). For applications of conjugated thio­phenes, see: Amaresh *et al.* (2002 ▶); Nielsen & Bjonholm (2004 ▶). For related structures, see: Dufresne *et al.* (2007 ▶); Kuroda *et al.* (2005 ▶).
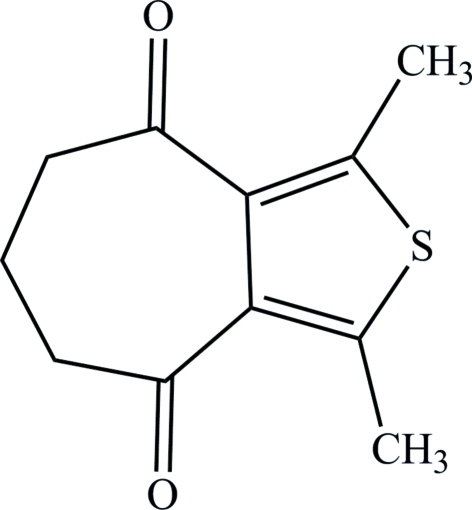


## Experimental

### 

#### Crystal data

C_11_H_12_O_2_S
*M* *_r_* = 208.27
Orthorhombic, 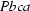

*a* = 15.9875 (6) Å
*b* = 7.6354 (3) Å
*c* = 16.9963 (6) Å
*V* = 2074.75 (13) Å^3^
*Z* = 8
Mo *K*α radiation
μ = 0.28 mm^−1^
*T* = 298 K
0.30 × 0.20 × 0.18 mm

#### Data collection

Bruker APEXII CCD area-detector diffractometer
Absorption correction: multi-scan (*SADABS*; Sheldrick, 2004 ▶) *T*_min_ = 0.920, *T*_max_ = 0.951
15732 measured reflections
1822 independent reflections
1430 reflections with *I* > 2σ(*I*)
*R*_int_ = 0.034

#### Refinement

*R*[*F* ^2^ > 2σ(*F* ^2^)] = 0.039
*wR*(*F* ^2^) = 0.109
*S* = 1.02
1822 reflections
129 parameters
H-atom parameters constrained
Δρ_max_ = 0.16 e Å^−3^
Δρ_min_ = −0.29 e Å^−3^

### 

Data collection: *APEX2* (Bruker, 2004 ▶); cell refinement: *SAINT-Plus* (Bruker, 2001 ▶); data reduction: *SAINT-Plus*; program(s) used to solve structure: *SHELXS97* (Sheldrick, 2008 ▶); program(s) used to refine structure: *SHELXL97* (Sheldrick, 2008 ▶); molecular graphics: *SHELXTL-Plus* (Sheldrick, 2008 ▶); software used to prepare material for publication: *SHELXL97*.

## Supplementary Material

Crystal structure: contains datablocks I, global. DOI: 10.1107/S1600536810047331/vm2059sup1.cif

Structure factors: contains datablocks I. DOI: 10.1107/S1600536810047331/vm2059Isup2.hkl

Additional supplementary materials:  crystallographic information; 3D view; checkCIF report

## Figures and Tables

**Fig. 1.**
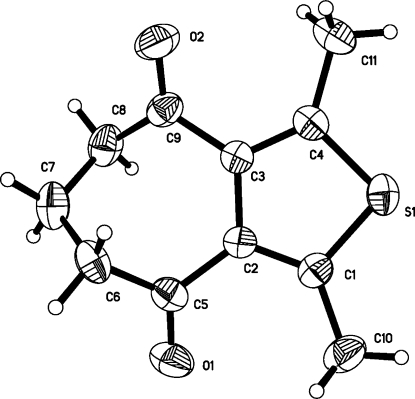
The molecular structure of (I), with atom labels and 30% probability displacement ellipsoids for non-H atoms.

**Fig. 2.**
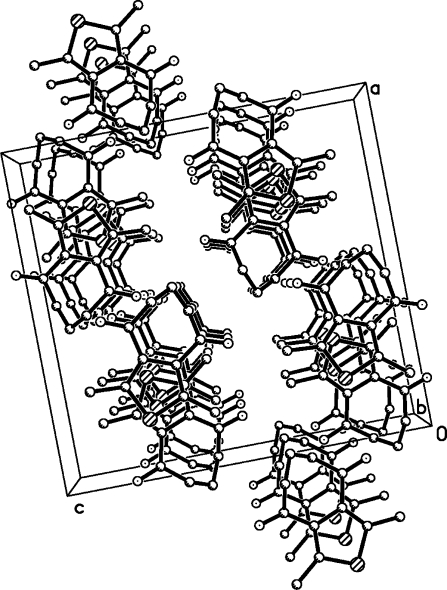
The packing of (I) viewed along the b-direction.

## supplementary crystallographic information

### Comment

Since the sulfur atom can contribute to the peripheral conjugation either by two *p*-electron moieties with its lone pair electrons or by *p*-sulfurane type conjugation, conjugated thiophenes have received much attention because of many new possibilities for constructing devices displaying unique optical, electrical, and mechanical properties (Nielsen *et al.*, 2004). Certain applications of conjugated thiophenes involve organic light emitting diodes and molecular wires, to be used in flexible light displays and/or low power consumption products (Amaresh *et al.*, 2002). Here, we report the structure of a novel conjugated thiophenes.

The crystal structure of the title compound is given in Fig.1. The crystallographic analysis confirms that the title compound consists of a central thiophene capped by two methyl groups. The molecular symmetry can be described by point group C2. The cycloheptane ring shows a twisted boat conformation. The C—C bond lengths with each methyl are almost equal, with an average value of 1.506 (3) Å. Futhermore, π-π interactions stabilize the packing (Fig. 2). The closest centroid distance of approximate paraller thiophene rings is 3.9759 (10) Å.

### Experimental

The title compound was prepared according to the literature (Kuroda *et al.*, 2005), using diffusion of hexane into a toluene solution of the title compound at room temperature. ^1^H NMR (CDCl_3_, δ, p.p.m.): 2.45 (m, 4H), 2.32 (s, 6H), 1.93 (m, 2H). Analysis calculated (%): C 63.43, H 5.81; found (%): C 63.20, H 6.05.

### Refinement

All H-atoms were positioned geometrically and constrained to ride on their parent atoms, with C—H = 0.96 and 0.97 Å, and with *U*_iso_(H) = 1.2*U*_eq_(C) or *U*_iso_(H) = 1.5*U*_eq_(C) for methyl H atoms.

### Crystal data

**Table:**

C_11_H_12_O_2_S	*F*(000) = 880
*M**_r_* = 208.27	*D*_x_ = 1.333 Mg m^−^^3^
Orthorhombic, *P**b**c**a*	Mo *K*α radiation, λ = 0.71073 Å
Hall symbol: -P 2ac 2ab	Cell parameters from 4563 reflections
*a* = 15.9875 (6) Å	θ = 2.4–22.2°
*b* = 7.6354 (3) Å	µ = 0.28 mm^−^^1^
*c* = 16.9963 (6) Å	*T* = 298 K
*V* = 2074.75 (13) Å^3^	Block, yellow
*Z* = 8	0.30 × 0.20 × 0.18 mm

### Data collection

**Table:**

Bruker APEXII CCD area-detector diffractometer	1822 independent reflections
Radiation source: fine-focus sealed tube	1430 reflections with *I* > 2σ(*I*)
graphite	*R*_int_ = 0.034
phi and ω scans	θ_max_ = 25.0°, θ_min_ = 2.4°
Absorption correction: multi-scan (*SADABS*; Sheldrick, 2004)	*h* = −16→19
*T*_min_ = 0.920, *T*_max_ = 0.951	*k* = −9→8
15732 measured reflections	*l* = −20→20

### Refinement

**Table:**

Refinement on *F*^2^	Primary atom site location: structure-invariant direct methods
Least-squares matrix: full	Secondary atom site location: difference Fourier map
*R*[*F*^2^ > 2σ(*F*^2^)] = 0.039	Hydrogen site location: inferred from neighbouring sites
*wR*(*F*^2^) = 0.109	H-atom parameters constrained
*S* = 1.02	*w* = 1/[σ^2^(*F*_o_^2^) + (0.0555*P*)^2^ + 0.5349*P*] where *P* = (*F*_o_^2^ + 2*F*_c_^2^)/3
1822 reflections	(Δ/σ)_max_ < 0.001
129 parameters	Δρ_max_ = 0.16 e Å^−^^3^
0 restraints	Δρ_min_ = −0.29 e Å^−^^3^

### Special details

**Table:**

Geometry. All e.s.d.'s (except the e.s.d. in the dihedral angle between two l.s. planes) are estimated using the full covariance matrix. The cell e.s.d.'s are taken into account individually in the estimation of e.s.d.'s in distances, angles and torsion angles; correlations between e.s.d.'s in cell parameters are only used when they are defined by crystal symmetry. An approximate (isotropic) treatment of cell e.s.d.'s is used for estimating e.s.d.'s involving l.s. planes.
Refinement. Refinement of *F*^2^ against ALL reflections. The weighted *R*-factor *wR* and goodness of fit *S* are based on *F*^2^, conventional *R*-factors *R* are based on *F*, with *F* set to zero for negative *F*^2^. The threshold expression of *F*^2^ > σ(*F*^2^) is used only for calculating *R*-factors(gt) *etc*. and is not relevant to the choice of reflections for refinement. *R*-factors based on *F*^2^ are statistically about twice as large as those based on *F*, and *R*- factors based on ALL data will be even larger.

### Fractional atomic coordinates and isotropic or equivalent isotropic displacement parameters (Å^2^)

**Table:**

	*x*	*y*	*z*	*U*_iso_*/*U*_eq_	
S1	0.20027 (4)	0.11743 (8)	0.72032 (3)	0.0670 (2)	
C2	0.35875 (12)	0.1124 (2)	0.70942 (10)	0.0496 (5)	
C3	0.32681 (12)	0.1405 (2)	0.63092 (10)	0.0493 (5)	
C1	0.29639 (14)	0.0980 (3)	0.76406 (11)	0.0575 (5)	
C9	0.37901 (14)	0.1832 (3)	0.56162 (11)	0.0614 (5)	
O2	0.35621 (12)	0.1474 (2)	0.49522 (8)	0.0898 (6)	
C4	0.24140 (14)	0.1457 (2)	0.62814 (11)	0.0557 (5)	
O1	0.47614 (12)	0.0907 (2)	0.79362 (10)	0.0882 (5)	
C5	0.44794 (14)	0.0756 (3)	0.72741 (12)	0.0593 (5)	
C6	0.50182 (13)	0.0112 (3)	0.66046 (14)	0.0688 (6)	
H6A	0.4706	−0.0746	0.6303	0.083*	
H6B	0.5508	−0.0467	0.6819	0.083*	
C11	0.18386 (16)	0.1778 (3)	0.55955 (15)	0.0779 (7)	
H11A	0.2030	0.2778	0.5305	0.117*	
H11B	0.1282	0.1991	0.5785	0.117*	
H11C	0.1836	0.0768	0.5259	0.117*	
C10	0.30152 (17)	0.0610 (4)	0.85104 (12)	0.0842 (7)	
H10A	0.3411	−0.0312	0.8602	0.126*	
H10B	0.2475	0.0258	0.8700	0.126*	
H10C	0.3192	0.1648	0.8782	0.126*	
C8	0.45990 (15)	0.2776 (3)	0.57771 (14)	0.0766 (7)	
H8A	0.4501	0.3667	0.6174	0.092*	
H8B	0.4778	0.3364	0.5300	0.092*	
C7	0.53003 (16)	0.1575 (3)	0.60577 (16)	0.0838 (7)	
H7A	0.5568	0.1056	0.5601	0.101*	
H7B	0.5716	0.2277	0.6328	0.101*	

### Atomic displacement parameters (Å^2^)

**Table:**

	*U*^11^	*U*^22^	*U*^33^	*U*^12^	*U*^13^	*U*^23^
S1	0.0548 (4)	0.0684 (4)	0.0779 (4)	0.0012 (3)	0.0116 (3)	−0.0081 (3)
C2	0.0545 (12)	0.0435 (10)	0.0509 (10)	0.0014 (8)	−0.0025 (8)	−0.0017 (8)
C3	0.0536 (12)	0.0423 (10)	0.0520 (10)	0.0016 (8)	−0.0011 (8)	−0.0048 (8)
C1	0.0666 (14)	0.0511 (12)	0.0549 (11)	0.0028 (9)	0.0053 (9)	−0.0034 (8)
C9	0.0753 (15)	0.0547 (12)	0.0542 (12)	0.0090 (11)	0.0068 (10)	0.0051 (9)
O2	0.1081 (14)	0.1103 (15)	0.0510 (9)	0.0094 (11)	0.0021 (8)	0.0020 (8)
C4	0.0569 (13)	0.0482 (11)	0.0620 (11)	0.0026 (9)	−0.0061 (9)	−0.0085 (8)
O1	0.0824 (12)	0.1029 (13)	0.0792 (11)	0.0074 (10)	−0.0269 (9)	0.0002 (9)
C5	0.0603 (13)	0.0496 (11)	0.0679 (12)	−0.0008 (10)	−0.0089 (10)	0.0025 (9)
C6	0.0524 (13)	0.0577 (13)	0.0962 (16)	0.0081 (10)	−0.0042 (11)	−0.0077 (11)
C11	0.0685 (15)	0.0810 (16)	0.0841 (16)	0.0095 (12)	−0.0247 (12)	−0.0115 (13)
C10	0.105 (2)	0.0922 (17)	0.0554 (13)	0.0103 (15)	0.0135 (12)	0.0038 (12)
C8	0.0785 (16)	0.0637 (14)	0.0875 (16)	−0.0077 (13)	0.0204 (13)	0.0109 (12)
C7	0.0628 (15)	0.0810 (17)	0.1076 (18)	−0.0070 (13)	0.0210 (14)	−0.0048 (14)

### Geometric parameters (Å, °)

**Table:**

S1—C4	1.713 (2)	C6—H6A	0.9700
S1—C1	1.714 (2)	C6—H6B	0.9700
C2—C1	1.367 (3)	C11—H11A	0.9600
C2—C3	1.445 (2)	C11—H11B	0.9600
C2—C5	1.485 (3)	C11—H11C	0.9600
C3—C4	1.367 (3)	C10—H10A	0.9600
C3—C9	1.480 (3)	C10—H10B	0.9600
C1—C10	1.507 (3)	C10—H10C	0.9600
C9—O2	1.217 (2)	C8—C7	1.525 (4)
C9—C8	1.506 (3)	C8—H8A	0.9700
C4—C11	1.505 (3)	C8—H8B	0.9700
O1—C5	1.218 (2)	C7—H7A	0.9700
C5—C6	1.509 (3)	C7—H7B	0.9700
C6—C7	1.521 (3)		
			
C4—S1—C1	93.64 (10)	C4—C11—H11A	109.5
C1—C2—C3	112.43 (18)	C4—C11—H11B	109.5
C1—C2—C5	123.04 (18)	H11A—C11—H11B	109.5
C3—C2—C5	123.89 (17)	C4—C11—H11C	109.5
C4—C3—C2	112.91 (17)	H11A—C11—H11C	109.5
C4—C3—C9	121.98 (17)	H11B—C11—H11C	109.5
C2—C3—C9	124.64 (18)	C1—C10—H10A	109.5
C2—C1—C10	129.9 (2)	C1—C10—H10B	109.5
C2—C1—S1	110.63 (15)	H10A—C10—H10B	109.5
C10—C1—S1	119.38 (16)	C1—C10—H10C	109.5
O2—C9—C3	121.3 (2)	H10A—C10—H10C	109.5
O2—C9—C8	122.2 (2)	H10B—C10—H10C	109.5
C3—C9—C8	116.45 (18)	C9—C8—C7	113.58 (19)
C3—C4—C11	129.9 (2)	C9—C8—H8A	108.8
C3—C4—S1	110.39 (14)	C7—C8—H8A	108.8
C11—C4—S1	119.62 (18)	C9—C8—H8B	108.8
O1—C5—C2	121.9 (2)	C7—C8—H8B	108.8
O1—C5—C6	121.1 (2)	H8A—C8—H8B	107.7
C2—C5—C6	117.02 (17)	C6—C7—C8	114.50 (19)
C5—C6—C7	113.00 (18)	C6—C7—H7A	108.6
C5—C6—H6A	109.0	C8—C7—H7A	108.6
C7—C6—H6A	109.0	C6—C7—H7B	108.6
C5—C6—H6B	109.0	C8—C7—H7B	108.6
C7—C6—H6B	109.0	H7A—C7—H7B	107.6
H6A—C6—H6B	107.8		
			
C1—C2—C3—C4	−0.3 (2)	C9—C3—C4—C11	5.3 (3)
C5—C2—C3—C4	170.75 (18)	C2—C3—C4—S1	0.23 (19)
C1—C2—C3—C9	171.89 (18)	C9—C3—C4—S1	−172.24 (14)
C5—C2—C3—C9	−17.0 (3)	C1—S1—C4—C3	−0.06 (15)
C3—C2—C1—C10	177.1 (2)	C1—S1—C4—C11	−177.88 (17)
C5—C2—C1—C10	5.9 (3)	C1—C2—C5—O1	−26.3 (3)
C3—C2—C1—S1	0.3 (2)	C3—C2—C5—O1	163.48 (19)
C5—C2—C1—S1	−170.90 (15)	C1—C2—C5—C6	151.22 (19)
C4—S1—C1—C2	−0.14 (16)	C3—C2—C5—C6	−19.0 (3)
C4—S1—C1—C10	−177.35 (18)	O1—C5—C6—C7	−103.5 (2)
C4—C3—C9—O2	−34.1 (3)	C2—C5—C6—C7	78.9 (2)
C2—C3—C9—O2	154.3 (2)	O2—C9—C8—C7	−102.1 (3)
C4—C3—C9—C8	144.01 (19)	C3—C9—C8—C7	79.8 (2)
C2—C3—C9—C8	−27.6 (3)	C5—C6—C7—C8	−49.7 (3)
C2—C3—C4—C11	177.76 (19)	C9—C8—C7—C6	−37.6 (3)
